# Dietary patterns of Brazilian farmers and their relation with sociodemographic, labor, and lifestyle conditions

**DOI:** 10.1186/s12937-020-00542-y

**Published:** 2020-03-24

**Authors:** Monica Cattafesta, Glenda Blaser Petarli, Tamires Conceição da Luz, Eliana Zandonade, Olívia Maria de Paula Alves Bezerra, Luciane Bresciani Salaroli

**Affiliations:** 1 Graduate Program in Collective Health, Federal University of Espírito Santo, Vitória, Brazil; 2 Graduate Program in Nutrition and Health, Federal University of Espírito Santo, Vitória, Brazil; 3 Department of Family Medicine, Mental and Collective Health, Federal University of Ouro Preto, Ouro Preto, Brazil; 4 Health Sciences Center, Federal University of Espírito Santo, Av. Marechal Campos, 1468 - Campus Maruípe, Vitória, ES 29.040-090 Brazil

**Keywords:** Feeding behavior, Food consumption, Dietary pattern, Workers, Farmers

## Abstract

**Background:**

The eating habits have changed in the last few decades, but few studies prioritize the food consumption of farmers and the rural population. Therefore, the objective of this study was explore the sociodemographic, occupational and lifestyle factors to the high adherence these dietary patterns.

**Methods:**

This is a cross-sectional epidemiological study of 740 farmers (51.5%, *n* = 381 males; 48.5%, *n* = 359 females) from a municipality in Southeastern Brazil. Food intake data were obtained by applying multipass 24-h recall and dietary intake was presented in dietary patterns determined by Principal Component Analysis with varimax orthogonal rotation.

**Results:**

Three dietary patterns were identified. The first pattern, “local traditional”, was associated with sociodemographic and labor variables, being considered typical of the region’s farmer as white race/color (*p* = 0.003), not extra-physical activity (*p* = 0.014) and cultivating 5 or more crops (*p* = 0.005). The permanence of a “traditional Brazilian” pattern and the occurrence of an “industrialized” pattern were also observed. Farmers working in non-conventional agriculture were 54% less adhere to “traditional Brazilian” pattern (OR 0.46, 95% CI 0.25–0.86, *p* = 0.014). Individuals aged 50 and over years were 82% less likely (OR 0.18, 95% CI 0.10–0.30) to adhere to “industrialized” pattern. Still, individuals of lower socioeconomic class were 52% less likely to adhere to this pattern (OR 0.48, 95% CI 0.24–0.96). Farmers who spent R$ 200 or more *per capita* to buy food were more than twice as likely to adhere to this food pattern (OR 2.22, 95% CI 1.32–3.73), and who had the habit of frequently eating out were 1.62 as likely adhere to “industrialized” pattern (95% CI 1.11–2.36).

**Conclusions:**

The findings indicate changes in dietary patterns in rural areas of the country, maintaining a traditional Brazilian pattern, as well as a local and an industrialized pattern. This last pattern demonstrates that the contemporary rural population also opts for a diet with ultra-processed products, being associated with the characteristic habits of a more urbanized rural region.

## Background

Agriculture employs 1.3 billion people worldwide, representing about 40% of the global workforce [1], reaching 75% of occupations in low-income countries [2]. Agricultural productivity is essential to sustaining the nutritional and health status of billions of people [3], and small farmers are critical to the global food supply [4]. However, agriculture is a dangerous profession [5] and with priority of attention [6], due to its occupational health risks and the presence of non-communicable chronic diseases and their risk factors [7–11].

With regards to dietary pattern, the eating habits of Brazilians have been undergoing changes in recent decades, especially the decrease in the consumption of legumes, roots and tubers and higher relative consumption of meat, milk, sugar and soft drinks and other ultra-processed foods [12], higher expenses with food away from home [13] and continued growth in the purchase of ready-to-eat products and significant reduction in the share of food and culinary ingredients [14]. In Brazilian food surveys, some differences were identified for the rural area, presenting, in general, higher prevalence of micronutrient inadequacy [15], lower consumption of food away from home [16] and higher consumption of beans [16, 17].

Despite the finding of an increase in the morbidity and mortality profile of this population [7–11], representative research on the food consumption of farmers and the rural population is still scarce [18], especially studies developed with the purpose of studying the dietary patterns of these populations and their associated factors [4–6, 19–29].

In this scenario, the objective of this paper was explore the sociodemographic, occupational and lifestyle factors to the high adherence these dietary patterns.

## Methods

### Study design

This is an epidemiological study of cross-sectional and quantitative design developed in the municipality of Santa Maria de Jetibá, located in the highlands of the state of Espírito Santo, Southeastern Brazil. This study integrates, in larger scope, the study “Health condition and associated factors: a study in farmers of Espírito Santo - AgroSaúdES”.

The representative sample of male and female farmers exposed here met the following inclusion criteria: adults 18–59 years old, non-pregnant, who had agriculture as their main source of income and were in full employment for at least 6 months. To identify eligible farmers, we used data available in the records of individuals and families conducted by the Family Health Strategy teams, responsible for covering 100% of the eleven health regions of the municipality.

### Sampling

Seven thousand two hundred eighty-seven farmers were identified from a total of 4018 families. About this population universe, we calculated a minimum sample of 708 farmers, considering a sampling error of 3.5%, a 95% level of significance and prevalence of 50% to maximize sample [30]. In order to reach the minimum sample and considering possible losses, recruitment included 806 individuals.

To define the sample universe one list was built with the survey of the registration of individuals and families by the Community Health Agents, through the data available in the family register used by the Family Health Strategy teams. This register covers 100% of the eleven health regions of the municipality. At the time of the sample (September/2016), 4018 families were enrolled, with 7287 farmers. The participants were selected by stratified draw, proportionality the number of families per health region, in order to respect proportionality among the regions. In families with more than one eligible, only one individual was drawn, avoiding thus the interdependence of information. In cases of refusal of participation or non-attendance in data collection, a new participant in the waiting list of the lottery was called, respecting the sex and region of origin of the dropout.

### Data collect

Data collection took place between December 2016 and April 2017 in the facilities of the municipal health units. A semi-structured questionnaire containing questions about socioeconomic, occupational, lifestyle and food consumption characteristics was applied.

### Food consumption analysis

Food intake data were obtained by applying three 24-h recalls (R24h) during the interview, 2 days of the week and 1 day of the weekend within 15 days after the first R24h in the return interviews. In order to obtain greater accuracy of the portions consumed, photo albums were used to facilitate the identification and quantification of the consumed items. Data were collected from 790 farmers, but 27 individuals were excluded since they underwent only one R24h and 23 since they underwent only two (6.3% loss), resulting in a final sample of 740 farmers. As such, the total lied above the minimum sample of 708 farmers (Additional file 1).

The nutritional composition of the R24h was performed using the software *AvaNutri* 4.1, in which the Brazilian Table of Food Composition [31] was selected for extraction of nutritional information. Information from the manufacturer or from standard recipes was used for the registration of typical regional foods that were not available at a table and possible dietary supplements were registered. After the registration of the food and acquiring the calories, no exclusion was performed due to extremes in energy consumption [32].

Then, 355 different food items reported in the R24h were listed in order to identify eating patterns through Principal Component Analysis (PCA). From these items, 65 foods were removed for not constituting the eating habits of the analyzed population (consumption < 5% of the sample) [33, 34]. The remaining foods were allocated to 25 groups (Table 1), according to their nutritional characteristics and Pearson’s correlation between their food items [30, 34].

**Table 1:** Food Group Table according to nutritional characteristics and Pearson correlation used in Principal Component Analysis

Group	Foods
G1	Rice
G2	Beans
G3	Pasta
G4	Potatoes, yams and cassava
G5	Flour (flour and *farofa*)
G6	*Polenta* (*poleta* and corn-grits)
G7	White meat (poutries and fishes)
G8	Red meat (beef and pork)
G9	Eggs
G10	Soups and broths
G11	Oils and fats
G12	Vegetables (tomato, green condiment, lettuce, cabbage, cucumber, carrot, chayote, pepper, okra, green leafy vegetables, scarlet eggplant, pumpkin and pod)
G13	Coffee
G14	Sugar
G15	Industrialized breads, cookies, toasts and threads
G16	Homemade breads, *brote*^a^, cakes and cookies
G17	Butter and margarine
G18	Milk, cheese and yogurt
G19	Sausage, canned food, industrialized condiment and sauce
G20	Snacks, fried food, hamburger, hot dog, garlic bread and trooper’s beans
G21	Fruits (banana, lemon, apple, guava, mango, grape, watermelon and peach)
G22	Candies (chocolate, pies, jams, ice cream and other sweets)
G23	Juice and sugary beverages
G24	Soda
G25	Alcoholic drinks (distilled beverages, bear and wine)

Pearson Correlation Aggregation and nutritional characteristics. ^a^It is a typical homemade bread made with tubers and roots. Subtitle*: G* group

The applicability of the PCA method was evaluated by the Kaiser-Meyer-Olkin coefficient (KMO) and Bartlett’s test of sphericity (BTS). In this study, the KMO value was 0.609 and the BTS test *p-*value was *p* < 0.001, which indicates the adequacy of the data to the factor analysis and recommends its application to the group of farmers studied [35].

The number of factors retained was defined according to the following criteria: components with eigenvalues greater than 1.0; Cattel chart; and conceptual meaning of the identified patterns. After Cattel graph analysis, three factors were extracted during analysis based on the line’s inflection point on the graph [35]. Factor analysis was subsequently applied to the 25 food groups, selecting varimax rotation to obtain uncorrelated factors [35].

Foods or food groups whose factor saturation charges were above 0.3 were evaluated as having a strong association with the component, providing better information for the description of a dietary pattern [30, 34]. The patterns were named in accordance with the interpretability and characteristics of the items retained in each pattern, and the items with the highest factor loadings were the ones that most influenced the interpretation and denomination of factors [30, 34, 35].

### Independent variables

The independent variables of this study were subdivided into sociodemographic, labor, and lifestyle variables. Among the sociodemographic variables were evaluated sex, age group (“up to 29 years”, “30 to 39 years”, “40 to 49 years” and “50 years or more”), marital status (“single”, “married/living with a partner” and “divorced/separated/widowed”), race/color (“white” and “non-white”), land bond (“owner” and “non-owner”), schooling (“less than 4 years”, “4 to 8 years” and “more than 8 years”), transport used most frequently (“own vehicle” and “on foot, by bicycle or bus”), nearby places for physical activity (“there is no proper place” and “around the house”), and socioeconomic class (“A or B”, “C” and “D or E”) [36]. This classification is used in national studies and estimates socioeconomic classes according to the purchasing power of individuals and families, and also allows estimating the average monthly gross family income (A: approximately R$ 11,037.00; B: approximately R$ 6006.00; C: approximately R$ 1865.00; D/E: approximately R$ 895.00) [36].

Labor variables were investigated by questioning working time as a farmer (“under 10 years”, “from 10 to 29 years” and “30 years or more”), the current type of production (“conventional” and “non-conventional”), the number of worked crops (“up to 4 crops” and “5 or more crops”), the type of worked crops categorized into “temporary only”, “permanent only” and “temporary and permanent”, according to criteria of the Brazilian Institute of Geography and Statistics [37], the workload (hours/week) (“less than or equal to 40 hours” and “more than 40 hours”) and contact with pesticides (“direct contact” and “indirect contact, organic or agroecological”) [38].

Lifestyle variables included alcohol consumption, categorized as “non-drinking” and “drinking”; smoking, assessed according to the Smoker Approach and Treatment Consensus and categorized as “non-smoker” and “current and past smoker”; practice of physical activity extra-field (“yes” or “no”); screen time obtained by the sum of daily activities for television, video game and computer/cell phone, divided by the days of the week, classified as “no sedentary leisure” when < 2 h/day and “with sedentary leisure” when ≥ 2 h/day [39]. Also evaluated were the number of places where they usually buy food (“2 places or less” and “3 places or more”), the frequency of food purchases (“twice/month or more” and “once/month or less”), travel time to purchase food (“up to 15 minutes”, “16 to 29 minutes” and “more than 30 minutes”), monthly *per capita* expenditure on food purchases (“R$ 100 or less”, “> R$ 100 to < R$ 200” and “R$ 200 or more”), the habit of eating away from home (“no or rarely” and “yes, often”) and the place where they usually meal (“at a table” and “under a different setting”).

### Statistical analysis

The normality of the variables was assessed by the Shapiro-Wilk test. To describe the study variables, the median (50p) was used as a measure of central tendency, and the interquartile range (IQR) as a dispersion measure for continuous variables, and absolute and percentage values ​​for categorical variables. Regarding the association tests between the independent variables and the outcome for the qualitative variables, Pearson’s Chi-square test was used. When the expected values ​​in the table cells were less than five or when the sum of the column value was less than twenty, Fisher’s exact test was used. To analyze the association between a quantitative and a qualitative variable, due to the abnormality of the variables, the Mann-Whitney U test was used. When the qualitative variable had three or more categories, the Kruskal-Wallis test and the Mann-Whitney U test were performed two by two to identify the differences. Missing data were maintained due to low data loss, different number of individuals in each variable were reported in the table captions.

The binary logistic regression model was applied to assess the association between independent variables and adherence to dietary patterns (adherence below the median *versus* adherence above the median). Variables that were statistically significant with dietary patterns of up to 20% in the association analyses were tested in multiple models and adjusted for sex. We used the backward variable selection method with likelihood ratio test, adopting the model with the highest adjustment according to the Hosmer-Lemeshow test (*p* > 0.05, closer to 1.0). We also tested the assumptions of absence of multicollinearity (tolerance > 0.1 and variance inflation factor < 10), minimum sample size for the number of model variables (> 20 individuals per model variable and > 5 cases in each category of variables) and absence of outliers (absence of standardized residues > ± 3 standard deviations; up to 1% of standardized residues between ±2.5 and 3 standard deviations; and up to 5% of standardized residues between ±2.0 and 2.5 standard deviations, Cook’s distance < 1, and DFBeta < 1).

For all analyses, the level of significance adopted was α < 5% and these were performed using the statistical software IBM SPSS Statistics for Windows, version 22.0 (Armonk, NY: IBM Corp).

### Ethical issues

The study was approved by the Research Ethics Committee of the Health Sciences Center of the Federal University of Espírito Santo (Ufes), under number 1,856,331 (CAAE 52839116.3.0000.5060), and followed the precepts of the Declaration of Helsinki. All respondents signed the Informed Consent Form.

## Results

### General characteristics of study population

Most of the evaluated farmers were married or living with a partner, were in socioeconomic class C, had low schooling (67.7% with less than 4 years of schooling), 66.4% (*n* = 491) reported not having adequate place for physical activity and 93.5% (*n* = 690) used their own vehicle as means of transportation (Table 2). Almost 80% (78.1%, *n* = 578) owned their land and 90% (*n* = 666) worked in conventional agriculture. Half of the farmers (50.8%, *n* = 375) worked from 10 to 29 years in the field, 56.8% (*n* = 420) cultivated five or more crops (larger in men, *p* = 0.004), worked in temporary crop (43.2%, *n* = 320) and had a high workload (80.1% with more than 40 working hours per week, higher in men with *p* < 0.001). Almost 70% of farmers had direct contact with pesticides, this contact being greater in men (*p* < 0.001).

**Table 2:** Sociodemographic, labor and lifestyle characterization of farmers from a Brazilian region, by sex

Variables	Sex		*P*-value	Total
	Male	Female		n (%)
	n (%)	n (%)		
**Age group**			0.956	
Up to 29 years	101 (50.2)	100 (49.8)		201 (27.2)
30 to 39 years	114 (52.3)	104 (47.7)		218 (29.5)
40 to 49 years	93 (50.8)	90 (49.2)		183 (24.7)
50 years or more	73 (52.9)	65 (47.1)		138 (18.6)
**Marital status**			**< 0.001**	
Single	46 (82.1)	10 (17.9)		56 (7.6)
Married/living with a partner	321 (50.3)	317 (49.7)		638 (86.2)
Divorced/separated/widowed	14 (30.4)	32 (69.6)		46 (6.2)
**Race/color**^a^			0.212	
White	339 (50.7)	330 (49.3)		669 (90.4)
Non-white	42 (59.2)	29 (40.8)		71 (9.6)
**Socioeconomic class**			**< 0.001**	
A or B	41 (73.2)	15 (26.8)		56 (7.6)
C	208 (55.3)	168 (44.7)		376 (50.8)
D or E	132 (42.9)	176 (57.1)		308 (41.6)
**Land bond**^a^			0.155	
Owner	306 (52.9)	272 (47.1)		578 (78.1)
Non-owner	75 (46.3)	87 (53.7)		162 (21.9)
**Schooling**			0.655	
Less than 4 years	253 (50.5)	248 (49.5)		501 (67.7)
4 to 8 years	88 (54.7)	73 (45.3)		161 (21.8)
More than 8 years	40 (51.3)	38 (48.7)		78 (10.5)
**Transport used more frequently**^c^			**< 0.001**	
Own vehicle	369 (53.5)	321 (46.5)		690 (93.5)
On foot, by bicycle or bus	11 (22.9)	37 (77.1)		48 (6.5)
**Nearby places for physical activity**^a^			**< 0.001**	
There is no proper place	226 (59.3)	265 (73.8)		491 (66.4)
Around the house	155 (40.7)	94 (26.2)		249 (33.6)
**Working time as a farmer**^c^			0.756	
Under 10 years	20 (57.1)	15 (42.9)		35 (4.7)
From 10 to 29 years	194 (51.7)	181 (48.3)		375 (50.8)
30 years or more	166 (50.6)	162 (49.4)		328 (44.4)
**Current type of production**^a^			0.177	
Conventional	337 (50.6)	329 (49.4)		666 (90.0)
Non-conventional	44 (59.5)	30 (40.5)		74 (10.0)
**Number of worked crops**^a^			**0.004**	
Up to 4 crops	145 (38.1)	175 (48.7)		320 (43.2)
5 or more crops	236 (61.9)	184 (51.3)		420 (56.8)
**Type of worked crops**			0.415	
Temporary only	176 (49.3)	181 (50.7)		357 (48.2)
Permanent only	22 (48.9)	23 (51.1)		45 (6.1)
Temporary and permanent	183 (54.1)	155 (45.9)		338 (45.7)
**Workload (hours/week)**^a^			**< 0.001**	
Less than or equal to 40 h	40 (27.2)	107 (72.8)		147 (19.9)
More than 40 h	341 (57.5)	252 (42.5)		593 (80.1)
**Contact with pesticides**^a^			**< 0.001**	
Direct contact	327 (63.7)	186 (36.3)		513 (69.3)
Indirect contact, organic or agroecological	54 (23.8)	173 (76.2)		227 (30.7)
**Alcohol consumption**^a^			**< 0.001**	
Non-drinking	150 (35.8)	269 (64.2)		419 (56.6)
Drinking	231 (72.0)	90 (28.0)		321 (43.4)
**Smoking**^a^			**< 0.001**	
Non-smoker	283 (74.3)	346 (96.4)		629 (85.0)
Current and past smoker	98 (25.7)	13 (3.6)		111 (15.0)
**Physical activity extra-field**^a,b^			0.087	
No	303 (79.5)	303 (84.4)		606 (81.9)
Yes	78 (20.5)	56 (15.6)		134 (18.1)
**Screen time**			0.712	
No sedentary leisure	205 (50.7)	199 (49.3)		404 (54.6)
With sedentary leisure	175 (52.2)	160 (47.8)		335 (45.3)
**Number of places where they usually buy food**			0.269	
2 places or less	215 (53.3)	188 (46.7)		403 (54.5)
3 places or more	166 (49.3)	171 (50.7)		337 (45.5)
**Frequency of food purchases**^a^			0.251	
Twice/month or more	114 (55.1)	93 (44.9)		207 (28.0)
Once/month or less	267 (50.1)	266 (49.9)		533 (72.0)
**Travel time to purchase food**			0.131	
Up to 15 min	120 (57.7)	88 (42.3)		208 (28.1)
16 to 29 min	177 (49.4)	181 (50.6)		358 (48.4)
More than 30 min	81 (49.4)	83 (50.6)		164 (22.2)
**Monthly per capita expenditure on food purchases**^d^			0.436	
R$ 100 or less	138 (37.7)	141 (40.9)		279 (39.2)
> R$ 100 to < R$ 200	164 (44.8)	155 (44.9)		319 (44.9)
R$ 200 or more	64 (17.5)	49 (14.2)		113 (15.9)
**Habit of eating away from home**^a^			**< 0.001**	
No or rarely	212 (55.6)	282 (78.6)		494 (66.8)
Yes, often	169 (44.4)	77 (21.4)		246 (33.2)
**Place where they usually meal**^a^			0.738	
At the table	280 (73.5)	268 (74.7)		548 (74.1)
Under a different setting	101 (26.5)	91 (25.3)		192 (25.9)

Qui-quadrado Test. ^a^Fisher’s Exact Test. *n* = 740, ^b^*n* = 739, ^c^*n* = 738, ^d^*n* = 711

Regarding lifestyle (Table 2), 43.4% (*n* = 321) reported consuming alcohol and 15% (*n* = 111) reported being a current or former smoker, both more prevalent in males (*p* < 0.001). More than 80% didn’t practice any physical activity extra-field and 45.3% (*n* = 335) presented sedentary leisure assessed by screen time. Regarding eating habits, 54.5% (*n* = 403) purchased food from two or less different locations and purchased low-frequency food (72% once/month or less, *n* = 533). The amount spent *per capita* on food purchases in almost half of the farmers (44.9%, *n* = 319) ranged from over R$ 100 up to R$ 200 per month. The habit of eating out was frequently reported by 33.2% of the individuals (*n* = 246), with this practice being more common among men (*p* < 0.001) and eating at a table was present in 74.1% (*n* = 548) of farmers.

### Dietary sources and nutritional characteristics

The most consumed items by this population were rice and coffee, beans, poultry meat, sugar, butter and margarine, homemade bread, oils, pasta, tomato, potato, and others typically foods (Table 3). Tomato was the only vegetable consumed in more than half of the population (64.9%) and 88.6% of the farmers consume at least some vegetables. The other vegetables consumed were green condiment, lettuce, cabbage, cucumber, carrot, and others. Fruit consumption was even lower, only 48.9% of farmers consume some kind of fruit. The most consumed fruit was banana, followed by lemon, apple, guava, mango, grape, watermelon and peach.

**Table 3:** The most consumed items, vegetables and fruit by farmers from a Brazilian region

Food items	% (n)
**All foods**	
Coffee	99.2 (734)
Rice	99.2 (734)
Beans	97.6 (722)
Poultry meat	93.5 (692)
Sugar	93.1 (689)
Butter and margarine	87.7 (649)
Homemade bread	86.9 (643)
Oils	84.7 (627)
Pasta	78.1 (578)
Tomato	64.9 (480)
Potato	63.2 (468)
Pork	60.4 (447)
Lard	49.1 (363)
Beef	48.0 (355)
Eggs	45.3 (335)
Juice	43.8 (324)
Soda	43.7 (323)
Milk	39.1 (289)
Flour	38.5 (285)
**Vegetables**	
Tomato	64.9 (480)
Green condiment	32.8 (243)
Lettuce	30.1 (223)
Cabbage	25.4 (188)
Cucumber	20.8 (154)
Carrot	15.3 (113)
Chayote	13.1 (97)
Pepper	10 (74)
Okra	9.9 (73)
Green leafy vegetables	8.5 (63)
Scarlet eggplant	8.4 (62)
Pumpkin	5.3 (39)
Pod	5.1 (38)
**Fruit**	
Banana	14.7 (109)
Lemon	14.1 (104)
Apple	11.4 (84)
Guava	8.1 (60)
Mango	7.4 (55)
Grape	6.2 (46)
Watermelon	6.1 (45)
Peach	5.7 (42)

Relative and absolute frequency. *N* = 740

### Dietary patterns

After rotational factor analysis, three dietary patterns were obtained (Table 4), namely: “pattern 1 – local traditional”: sugar; coffee; butter and margarine; homemade bread, *brote*, cakes and cookies; juice and sugary beverages; potatoes, yams and cassava; and pasta; “pattern 2 – traditional Brazilian”: beans; rice; vegetables (tomato, green condiment, lettuce, cabbage, cucumber, carrot, chayote, pepper, okra, green leafy vegetables, scarlet eggplant, pumpkin and pod); flour (flour and *farofa*); and oils and fats; and “pattern 3 – industrialized”: soda; snacks, fried food, hamburger, hot dog, garlic bread and trooper’s beans; red meat (beef and pork); sausage, canned food, industrialized condiment and sauce; alcoholic drinks (distilled beverages, bear and wine); and industrialized breads, cookies, toasts and threads. The component “homemade breads, *brote*, cakes and cookies” presented high negative factor load in the “industrialized” group, which demonstrates that individuals of this first dietary pattern have very low consumption of this type of food.

**Table 4:** Factorial load distribution table of food/food groups of the three food patterns identified for farmers

Food items	Dietary pattern		
	1	2	3
	Local traditional	Traditional Brazilian	Industrialized
Sugar	**0.736**	0.121	0.085
Coffee	**0.608**	0.246	−0.087
Butter and margarine	**0.571**	0.200	−0.095
Homemade breads, *brote*, cakes and cookies	**0.459**	0.158	− 0.405
Juice and sugary beverages	**0.438**	0.016	0.162
Potatoes, yams and cassava	**0.367**	−0.070	0.019
Pasta	**0.332**	0.112	0.141
Beans	0.247	**0.705**	− 0.062
Rice	0.131	**0.695**	0.062
Vegetables (tomato, green condiment, lettuce, cabbage, cucumber, carrot, chayote, pepper, okra, green leafy vegetables, scarlet eggplant, pumpkin and pod)	− 0.164	**0.521**	−0.059
Flour (flour and *farofa*)	0.058	**0.479**	−0.039
Oils and fats	−0.048	**0.358**	−0.045
Soda	0.103	−0.058	**0.621**
Snacks, fried food, hamburger, hot dog, garlic bread and trooper’s beans	0.056	−0.064	**0.508**
Red meat (beef and pork)	−0.088	0.299	**0.490**
Sausage, canned food, industrialized condiment and sauce	0.104	0.023	**0.461**
Alcoholic drinks (distilled beverages, bear and wine)	−0.066	− 0.004	**0.449**
Industrialized breads, cookies, toasts and threads	−0.042	0.045	**0.374**
White meat (poutries and fishes)	0.285	−0.022	−0.049
Candies (chocolate, pies, jams, ice cream and other sweets)	0.122	−0.078	0.248
Eggs	0.099	0.230	−0.027
Soups and broths	0.031	−0.254	−0.074
Fruits (banana, lemon, apple, guava, mango, grape, watermelon and peach)	−0.046	− 0.018	0.074
Milk, cheese and yogurt	−0.122	0.032	0.078
*Polenta* (*poleta* and corn-grits)	−0.157	0.040	−0.023
*Accumulated explained variance*	*8.7*	*16.7*	*23.8*

Principal Component Analysis (PCA)

The total variance explained by the factors was 23.8%. Foods with a low factorial load in one component were considered to be of low correlation and did not saturated in the any dietary pattern, which makes it possible to consider them as foods of homogeneous consumption among individuals. They are: white meat (poutries and fishes), candies (chocolate, pies, jams, ice cream and other sweets), eggs, soups and broths, fruits (banana, lemon, apple, guava, mango, grape, watermelon and peach), milk, cheese and yogurt and *polenta*.

### Factors effect on farmers’ dietary pattern

Associated with the greater adherence to the “traditional local” pattern was the male sex, the age group from 30 to 39 years old, the race/white color, having 4 to 8 years of study, transport using their own vehicle, owning the land, working with 5 or more crops and more than 40 h per week, having direct contact with pesticides, consumption of alcoholic beverages and eating out frequently (Table 5). Lower adherence to this first pattern was associated with being separated, divorced or widowed and working only with permanent crops. Regarding the greater adherence to the “traditional Brazilian” pattern, men were associated with transport using their own vehicle, having direct contact with pesticides, consuming alcoholic beverages and being a current or former smoker. In addition, it was associated with greater adherence to the “industrialized” pattern to be male, aged up to 29 years old, single and non-white, socioeconomic class A or B, over 8 years old of schooling, have a place to practice physical activity in the residence surroundings, transport using their own vehicle, working only with temporary crops, having direct contact with pesticides, drink alcohol, be a current or former smoker, have sedentary leisure, buy food in 3 places or more, spend R$ 200 or more (*per capita*/month) on food, eating often outside, and eating away from a table.

**Table 5:** Binary analysis of adherence scores to dietary patterns according to sociodemographic, occupational and lifestyle characteristics

Variables	Pattern 1 – Local traditional	Pattern 2 – Traditional Brazilian	Pattern 3 – Industrialized
	50p (IQR)	50p (IQR)	50p (IQR)
**Sex**	***p < 0.001***	***p < 0.001***	***p < 0.001***
Male	0.22 (− 0.40–0.98)	0.14 (− 0.42–0.80)	0.04 (− 0.59–0.84)
Female	−0.43 (− 0.91–0.11)	− 0.36 (− 0.86–0.20)	− 0.36 (− 0.78–0.13)
**Age group**^g^	***p < 0.001***^***c.e.f***^	*p = 0.47*	***p < 0.001***^***a.b.c.e***^
Up to 29 years	− 0.03 (− 0.56–0.71)	− 0.17 (− 0.61–0.35)	0.11 (− 0.43–0.85)
30 to 39 years	0.04 (− 0.63–0.76)	− 0.01 (− 0.56–0.68)	− 0.04 (− 0.65–0.59)
40 to 49 years	− 0.18 (− 0.74–0.49)	− 0.02 (− 0.70–0.46)	− 0.36 (− 0.80–0.11)
50 years or more	−0.43 (− 0.94–0.23)	−0.12 (− 0.62–0.50)	−0.46 (− 0.88-(− 0.07))
**Marital status**^g^	***p = 0.021***^***b.c***^	*p = 0.791*	***p = 0.026***^***b.c***^
Single	−0.09 (− 0.61–0.92)	−0.09 (− 0.56–0.63)	0.07 (− 0.61–1.13)
Married/living with a partner	−0.12 (− 0.68–0.58)	−0.07 (− 0.60–0.48)	−0.16 (− 0.70–0.45)
Divorced/separated/widowed	−0.37 (−1.06–0.19)	−0.16 (− 0.81–0.42)	−0.42 (− 0.83-(− 0.06))
**Race/color**	***p = 0.002***	*p = 0.089*	***p = 0.001***
White	−0.09 (− 0.66–0.58)	−0.11 (− 0.63–0.46)	−0.23 (− 0.74–0.40)
Non-white	−0.49 (− 0.96–0.29)	0.05 (− 0.53–0.79)	0.03 (− 0.41–0.92)
**Socioeconomic class**^g^	*p = 0.399*	*p = 0.503*	***p < 0.001***^***a.b.c***^
A or B	− 0.33 (− 0.78–0.53)	−0.05 (− 0.38–0.47)	0.38 (− 0.40–1.13)
C	−0.08 (− 0.69–0.63)	−0.12 (− 0.66–0.46)	−0.09 (− 0.65–0.54)
D or E	−0.15 (− 0.68–0.43)	−0.06 (− 0.60–0.53)	−0.36 (− 0.78–0.20)
**Land bond**	***p = 0.013***	*p = 0.707*	*p = 0.495*
Owner	−0.07 (− 0.64–0.61)	−0.06 (− 0.65–0.54)	−0.20 (− 0.73–0.46)
Non-owner	−0.23 (− 0.91–0.36)	−0.14 (− 0.55–0.36)	−0.11 (− 0.60–0.47)
**Schooling**^g^	***p = 0.008***^***a***^	*p = 0.435*	***p < 0.001***^***a.b***^
Less than 4 years	−0.22 (− 0.78–0.44)	−0.06 (− 0.61–0.55)	−0.31 (− 0.78–0.29)
4 to 8 years	0.00 (− 0.56–0.75)	−0.20 (− 0.61–0.31)	0.08 (− 0.47–0.84)
More than 8 years	0.05 (− 0.58–0.78)	−0.01 (− 0.66–0.46)	0.13 (− 0.42–0.97)
**Transport used more frequently**^i^	***p = 0.006***	***p = 0.033***	***p = 0.013***
Own vehicle	−0.11 (− 0.68–0.58)	−0.06 (− 0.60–0.54)	−0.14 (− 0.70–0.50)
On foot, by bicycle or bus	−0.47 (− 1.02–0.12)	−0.38 (− 0.91–0.15)	−0.40 (− 0.71-(− 0.13))
**Nearby places for physical activity**	*p = 0.888*	*p = 0.122*	***p = 0.001***
There is no proper place	−0.12 (− 0.69–0.58)	−0.13 (− 0.63–0.43)	−0.28 (− 0.72–0.32)
Around the house	−0.17 (− 0.70–0.56)	−0.02 (− 0.61–0.64)	0.03 (− 0.67–0.70)
**Current type of production**	*p = 0.472*	*p = 0.090*	*p = 0.932*
Conventional	−0.14 (− 0.69–0.58)	−0.06 (− 0.63–0.54)	−0.19 (− 0.71–0.47)
Non-conventional	−0.17 (− 0.80–0.37)	−0.26 (− 0.57–0.24)	−0.21 (− 0.65–0.23)
**Number of worked crops**	***p < 0.001***	*p = 0.476*	*p = 0.552*
Up to 4 crops	−0.30 (− 0.82–0.35)	−0.14 (− 0.64–0.50)	−0.25 (− 0.70–0.45)
5 or more crops	0.00 (− 0.57–0.75)	−0.03 (− 0.60–0.48)	−0.14 (− 0.71–0.48)
**Type of worked crops**^g^	***p = 0.001***^***a.c***^	*p = 0.710*	***p = 0.007***^***a.b***^
Temporary only	−0.06 (− 0.62–0.61)	−0.13 (− 0.64–0.48)	−0.32 (− 0.77–0.34)
Permanent only	−0.48 (− 0.91-(− 0.03))	−0.14 (− 0.57–0.30)	−0.07 (− 0.60–0.82)
Temporary and permanent	−0.15 (− 0.77–0.53)	−0.04 (− 0.58–0.52)	−0.05 (− 0.64–0.50)
**Workload (hours/week)**	***p < 0.001***	*p = 0.296*	*p = 0.154*
Less than or equal to 40 h	−0.46 (− 1.04–0.05)	−0.20 (− 0.61–0.44)	−0.32 (− 0.69–0.28)
More than 40 h	−0.04 (− 0.61–0.70)	−0.06 (− 0.62–0.54)	−0.12 (− 0.71–0.48)
**Contact with pesticides**	***p < 0.001***	***p < 0.001***	***p = 0.008***
Direct contact	−0.06 (− 0.61–0.65)	0.00 (− 0.58–0.68)	−0.10 (− 0.65–0.51)
Indirect contact, organic or agroecological	−0.37 (− 0.82–0.24)	−0.28 (− 0.69–0.23)	−0.35 (− 0.76–0.23)
**Alcohol consumption**	***p = 0.036***	***p < 0.001***	***p < 0.001***
Non-drinking	−0.21 (− 0.78–0.49)	−0.20 (− 0.73–0.32)	−0.36 (− 0.78–0.20)
Drinking	−0.05 (− 0.63–0.61)	0.05 (− 0.53–0.76)	0.09 (− 0.54–0.75)
**Smoking**	*p = 0.112*	***p < 0.001***	*p = 0.059*
Non-smoker	− 0.12 (− 0.66–0.58)	−0.14 (− 0.67–0.38)	−0.23 (− 0.73–0.42)
Current and past smoker	−0.22 (− 0.92–0.41)	0.31 (− 0.42–0.89)	−0.01 (− 0.52–0.70)
**Physical activity extra-field**	*p = 0.185*	*p = 0.363*	***p < 0.001***
No	−0.11 (− 0.66–0.54)	−0.09 (− 0.58–0.50)	−0.26 (− 0.74–0.40)
Yes	−0.33 (− 0.85–0.72)	−0.06 (− 0.82–0.45)	0.06 (− 0.54–0.91)
**Screen time**^h^	*p = 0.654*	*p = 0.660*	***p = 0.001***
No sedentary leisure	−0.15 (− 0.69–0.51)	−0.08 (− 0.58–0.54)	−0.31 (− 0.79–0.30)
With sedentary leisure	−0.11 (− 0.71–0.64)	−0.10 (− 0.66–0.47)	−0.02 (− 0.59–0.53)
**Number of places where they usually buy food**	*p = 0.663*	*p = 0.689*	***p = 0.001***
2 places or less	−0.12 (− 0.66–0.55)	−0.11 (− 0.60–0.46)	−0.31 (− 0.79–0.29)
3 places or more	−0.17 (− 0.74–0.59)	−0.04 (− 0.64–0.52)	−0.07 (− 0.60–0.58)
**Frequency of food purchases**	*p = 0.269*	*p = 0.780*	*p = 0.493*
Twice/month or more	−0.21 (− 0.80–0.59)	−0.05 (− 0.57–0.46)	−0.13 (− 0.79–0.60)
Once/month or less	−0.11 (− 0.63–0.55)	−0.10 (− 0.64–0.50)	−0.22 (− 0.67–0.38)
**Travel time to purchase food**^g,j^	*p = 0.597*	*p = 0.957*	*p = 0.425*
Up to 15 min	−0.12 (− 0.67–0.67)	−0.07 (− 0.60–0.45)	−0.03 (− 0.73–0.79)
16 to 29 min	−0.21 (− 0.73–0.55)	−0.13 (− 0.63–0.55)	−0.22 (− 0.71–0.46)
More than 30 min	0.02 (−0.64–0.51)	−0.05 (− 0.58–0.45)	−0.20 (− 0.65–0.32)
**Monthly per capita expenditure on food purchases**^g,k^	*p = 0.206*	*p = 0.330*	***p < 0.001***^***a.b.c***^
R$ 100 or less	−0.04 (− 0.62–0.62)	−0.01 (− 0.54–0.45)	−0.31 (− 0.82–0.25)
> R$ 100 to < R$ 200	−0.17 (− 0.75–0.44)	−0.14 (− 0.67–0.45)	−0.11 (− 0.65–0.56)
R$ 200 or more	−0.28 (− 0.78–0.58)	−0.08 (− 0.64–0.74)	0.07 (− 0.46–0.80)
**Habit of eating away from home**	***p = 0.001***	*p = 0.847*	***p < 0.001***
No or rarely	−0.21 (− 0.76–0.42)	−0.07 (− 0.62–0.48)	−0.33 (− 0.79–0.25)
Yes, often	0.06 (− 0.61–0.80)	−0.13 (− 0.61–0.57)	0.07 (− 0.43–0.85)
**Place where they usually meal**	*p = 0.611*	*p = 0.170*	***p < 0.001***
At the table	−0.14 (− 0.66–0.55)	−0.11 (− 0.64–0.47)	−0.27 (− 0.78–0.38)
Under a different setting	−0.15 (− 0.84–0.66)	−0.03 (− 0.55–0.56)	0.04 (− 0.47–0.65)

Mann-Whitney U test. ^a^Difference between 1^st^ and 2^nd^ category, ^b^Difference between 1^st^ and 3^rd^ category, ^c^Difference between 2^nd^ and 3^rd^ category, ^d^Difference between 1^st^ and 4^th^ category, ^e^Difference between 2^nd^ and 4^th^ category, ^f^Difference between the 3^rd^ and 4^th^ category. ^g^Kruskal-Wallis test with Mann-Whitney U test two by two to identify differences. *N* = 740, ^h^*n* = 739, ^i^*n* = 738, ^j^*n* = 730, ^k^*n* = 711. Subtitle: *50p* median, *IQR* interquartile range (25 percentil to 75 percentil), *p p*-value

From the found associations, multiple analyses were performed for each dietary pattern (Tables 6, 7 and 8). Thus, the variables "age group, race/color, number of worked crops and physical activity extra-field were associated with the “local traditional” pattern (Table 6). Individuals aged 50 years and older were 56% less likely to adhere to this dietary pattern than those up to 29 years old (OR 0.44, 95% CI 0.25–0.74, *p* = 0.003). Farmers of non-white race/color were 58% less likely to be more adherent to this dietary pattern (OR 0.42, 95% CI 0.23–0.74, *p* = 0.003) and those who practiced extra-physical activity, 47% less likely to be more adherent to the “local traditional” pattern (OR 0.46, 95% CI 0.25–0.86, *p* = 0.014). However, workers who cultivated 5 or more crops were 1.59 times more likely to adhere to this pattern (OR 1.59, 95% CI 1.15–2.19, *p* = 0.005).

**Table 6:** Multiple analysis of socioeconomic, occupational and lifestyle factors associated with “local traditional” farmer dietary patterns

Variables	Crude		Adjusted	
	*p*-value	OR (95% CI)	*p*-value	OR (95% CI)
**1st pattern – Local traditional**				
**Age group**				
Up to 29 years		1		1
30 to 39 years	0.879	1.03 (0.70–1.52)	0.550	1.14 (0.74–1.76)
40 to 49 years	0.197	0.77 (0.51–1.15)	0.456	0.83 (0.51–1.35)
50 years or more	**< 0.001**	0.43 (0.28–0.68)	**0.003**	0.44 (0.25–0.75)
**Race/color**				
White		1		1
Non-white	**0.002**	0.44 (0.26–0.75)	**0.003**	0.42 (0.23–0.74)
**Land bond**				
Owner		1		1
Non-owner	**0.033**	0.68 (0.48–0.97)	0.114	0.72 (0.48–1.08)
**Schooling**				
Less than 4 years		1		1
4 to 8 years	**0.015**	1.56 (1.09–2.23)	0.058	1.51 (0.99–2.31)
More than 8 years	0.167	1.40 (0.87–2.26)	0.400	1.26 (0.73–2.18)
**Number of worked crops**				
Up to 4 crops		1		1
5 or more crops	**< 0.001**	1.82 (1.36–2.44)	**0.005**	1.59 (1.15–2.19)
**Workload (hours/week)**				
Less than or equal to 40 h		1		1
More than 40 h	**< 0.001**	2.45 (1.67–3.59)	0.095	1.44 (0.94–2.21)
**Alcohol consumption**				
Non-drinking		1		1
Drinking	0.064	1.32 (0.98–1.76)	0.085	0.74 (0.52–1.04)
**Smoking**				
Non-smoker		1		1
Current and past smoker	0.607	0.90 (0.60–1.35)	0.093	0.66 (0.41–1.07)
**Physical activity extra-field**				
No		1		1
Yes	0.127	0.75 (0.51–1.09)	**0.003**	0.53 (0.34–0.81)

Binary logistic regression with backward selection method: likelihood ratio test, adjusted for sex. *N* = 740. Subtitles: *OR* odds ratio, *95% CI* 95% Confidence Interval

Variables with *p* < 0.2 in the binary analyzes were entered in the initial model, except for “Contact with pesticide” due to multicollinearity with “Type of worked crops”: sex, age group, marital status, race/color, schooling, transport used more frequently, land bond, number of worked crops, type of worked crops, weekly workload, alcohol consumption, smoking, physical activity extra-field and eating out. Only variables kept in the backward selection are presented in the table. Hosmer-Lemeshow model fit quality = 0.946

**Table 7:** Multiple analysis of socioeconomic, occupational and lifestyle factors associated with “traditional Brazilian” farmer dietary patterns

Variables	Crude		Adjusted	
	*p*-value	OR (95% CI)	*p*-value	OR (95% CI)
**2nd pattern - Traditional Brazilian**				
**Race/color**				
White		1		1
Non-white	0.063	1.61 (0.97–2.65)	0.150	1.47 (0.87–2.47)
**Transport used more frequently**				
Own vehicle		1		1
On foot, by bicycle or bus	0.133	0.63 (0.35–1.15)	0.642	0.86 (0.46–1.62)
**Current type of production**				
Conventional		1		1
Non-conventional	**0.029**	0.58 (0.35–0.94)	**0.014**	0.46 (0.25–0.86)
**Contact with pesticides**				
Direct contact		1		1
Indirect contact, organic or agroecological	**0.001**	0.58 (0.42–0.79)	0.660	1.10 (0.72–1.67)
**Alcohol consumption**				
Non-drinking		1		1
Drinking	**0.006**	1.50 (1.12–2.02)	0.696	1.07 (0.77–1.48)
**Smoking**				
Non-smoker		1		1
Current and past smoker	**0.001**	2.05 (1.35–3.12)	0.127	1.43 (0.90–2.25)

Binary logistic regression with backward selection method: likelihood ratio test, adjusted for sex. *N* = 740. Subtitles: *OR* odds ratio, *95% CI* 95% Confidence Interval

Variables with *p* < 0.2 in the binary analyzes were included in the initial model: sex, race/color, transportation, type of production, contact with pesticides, alcohol consumption and smoking. Only variables kept in the backward selection are presented in the table. Hosmer-Lemeshow model fit quality = 0.992

**Table 8:** Multiple analysis of socioeconomic, occupational and lifestyle factors associated with “industrialized” farmer dietary patterns

Variables	Crude		Adjusted	
	*p*-value	OR (95% CI)	*p*-value	OR (95% CI)
**3th pattern – Industrialized**				
**Age group**				
Up to 29 years		1		1
30 to 39 years	**0.027**	0.64 (0.43–0.95)	**0.009**	0.56 (0.36–0.87)
40 to 49 years	**< 0.001**	0.37 (0.25–0.56)	**< 0.001**	0.33 (0.21–0.53)
50 years or more	**< 0.001**	0.23 (0.14–0.36)	**< 0.001**	0.18 (0.10–0.30)
**Race/color**				
White		1		1
Non-white	0.063	1.61 (0.97–2.65)	0.066	1.74 (0.96–3.13)
**Socioeconomic class**				
A or B		1		1
C	0.141	0.64 (0.36–1.16)	0.316	0.71 (0.37–1.38)
D or E	**0.001**	0.35 (0.19–0.63)	**0.037**	0.48 (0.24–0.96)
**Nearby places for physical activity**				
There is no proper place		1		1
Around the house	**0.001**	1.65 (1.21–2.24)	0.065	1.40 (0.98–2.00)
**Type of worked crops**				
Temporary only		1		1
Permanent only	0.165	1.56 (0.83–2.91)	0.843	1.08 (0.52–2.25)
Temporary and permanent	**0.006**	1.52 (1.13–2.06)	**0.012**	1.57 (1.11–2.24)
**Alcohol consumption**				
Non-drinking		1		1
Drinking	**< 0.001**	2.21 (1.64–2.97)	0.050	1.43 (1.00–2.05)
**Number of places where they usually buy food**				
2 places or less		1		1
3 places or more	**0.002**	1.60 (1.20–2.14)	**0.002**	1.71 (1.21–2.41)
**Monthly per capita expenditure on food purchases**				
R$ 100 or less		1		1
> R$ 100 to < R$ 200	**0.044**	1.39 (1.01–1.92)	0.088	1.38 (0.95–1.99)
R$ 200 or more	**0.001**	2.06 (1.32–3.23)	**0.003**	2.22 (1.32–3.73)
**Habit of eating away from home**				
No or rarely		1		1
Yes, often	**< 0.001**	2.21 (1.61–3.03)	**0.012**	1.62 (1.11–2.36)
**Place where they usually meal**				
At the table		1		1
Under a different setting	**< 0.001**	1.82 (1.30–2.54)	**0.028**	1.56 (1.05–2.31)

Binary logistic regression with backward selection method: likelihood ratio test, adjusted for sex. *N* = 740. Subtitles: *OR* odds ratio, *95% CI* 95% Confidence Interval

Variables with *p* < 0.2 in the binary analyzes were entered in the initial model, except for “Contact with pesticide” due to multicollinearity with “Type of worked crops”: sex, age group, marital status, race/color, socioeconomic class, location for physical activity, most commonly used transportation, type of crops worked, weekly workload, alcohol consumption, smoking, physical activity extra-field, screen time, number of places buying food, spending on food, habit of eating out and eating places. Only variables kept in the backward selection are presented in the table. Hosmer-Lemeshow model fit quality = 0.954

Regarding the “traditional Brazilian” pattern, farmers working in non-conventional agriculture were 54% less likely to adhere more to this dietary pattern (OR 0.46, 95% CI 0.25–0.86, *p* = 0.014) (Table 7).

For the third pattern, it was identified that the higher the age group, the lower the chances of farmers adhering to the “industrialized” pattern (Table 8). Individuals aged 30 to 39 years were 44% less likely (OR 0.56, 95% CI 0.36–0.87, *p* = 0.009), those aged 40 to 49 years were 67% less likely (OR 0.33, 95% CI 0.21–0.53, *p* < 0.001) and those aged 50 and over, 82% less likely (OR 0.18, 95% CI 0.10–0.30, *p* < 0.001) to adhere to this pattern than those aged 30 and under. Still, individuals of socioeconomic class D or E were 52% less likely to adhere to this pattern (OR 0.48, 95% CI 0.24–0.96, *p* = 0.037), whereas those who worked with temporary and permanent tillage had 1.57 times more likely to adhere to the “industrialized” pattern (OR 1.57, 95% CI 1.21–2.41, *p* = 0.002) than those working only on temporary tillage. Still, farmers who spent R$ 200 or more *per capita* to buy food were more than twice as likely to adhere to this food pattern (OR 2.22, 95% CI 1.32–3.73, *p* = 0.003). Likewise, those who had the habit of frequently eating out were 1.62 times more likely to adhere to this pattern (OR 1.62, 95% CI 1.11–2.36, *p* = 0.012) and those without the habit of eating at a table, 1.56 times more likely to adhere more to the “industrialized” pattern (OR 1.56, 95% CI 1.05–2.31, *p* = 0.028).

## Discussion

Three dietary patterns were identified in the sample of farmers studied: “local traditional”, “traditional Brazilian” and “industrialized”, which shows the eating habits of this group of workers. Such patterns represent the eating habits of this group, with the “local traditional” pattern being typical of the region’s farmer, associated with the white race/color, not within the extreme age group, working with many crops and lack of exercise extra-field. In addition, it was evident that the “traditional Brazilian” pattern, with beans, rice and vegetables, remains in the eating habits of this population, and this pattern is associated with the conventional type of production. An “industrialized” pattern was also identified, which demonstrates that the contemporary rural farmer also opts for a diet with processed products. This pattern was associated with a lower age range; the highest socioeconomic class; productivity with temporary and permanent crops; the largest number of locations and the highest monthly expenditure on food purchases; eating away from home and eating away from a table, typical of a more urbanized rural setting. A low consumption of fruits was also found for this population.

Determining food patterns in this population is an unprecedented finding, because of the method used to determine dietary patterns, highly recommended for approaching the actual behavior of the population studied [40, 41], and for extrapolated the combination of nutrients and antinutritional factors involved in the human diet [40]. In addition, can meet the scarcity of studies that investigate the food consumption of farmers or rural populations and their associated factors [15–29], especially regarding their labor characteristics [5].

As in the present study, Andrade et al. (2018) [27], in a survey of 34,003 individuals from the 2008/2009 Household Budget Survey (POF), showed three dietary patterns in the Brazilian population, with traditional meals, “typical Brazilian breakfast/tea” and the last one with “ultra-processed foods”. Nogueira et al. (2019) [34], in a study with metallurgists from Fortaleza/CE, Brazil, also identified three eating patterns: the first common northeastern, the second popular general and the third western. These patterns shows similarities with respect to the consumption of a traditional local standard, a traditional Brazilian and an industrialized one.

Other studies on the determination of dietary patterns in working classes have also shown consistency with the patterns found in farmers, with appropriate interpretations in their working environments, such as “vegetables, fruits, cereals and tubers”, “sweets and snacks” and “traditional and protein” in banking [30]. Likewise, the “traditional”, “fruit and vegetables”, “pastry” and “diet/light” patterns in civil servants [42] and the “healthy”, “western” and “traditional” in teachers [43].

Typical “breakfast” or “snack” foods stood out in the “traditional local” pattern, due to the continued habit of some families producing their own foods such as breads, cookies, pies and home-made cakes [44]. This first pattern elucidates the eating habits of the region’s typical white-colored farmer, colonist (Pomeranian peasant immigrant), who still produces many of their own foods [44] and doesn’t use their time in physical activities beyond those already arising from work in the field, in particular by working with many crops for olericulture [45].

In addition to the snack pattern, the “traditional Brazilian pattern” was also identified. This patter represents the typical Brazilian diet, reinforced by the high frequency of consumption of rice, coffee and beans in this population. This indicates that, as in other population studies in rural areas, typical foods remain in the eating habits of these individuals [16, 21, 23]. In Brazil, three quarters of the population regularly consume beans (71.9, 95% CI 71.2–72.6%), and the state of Espírito Santo is the place with the highest consumption of this food (86.5, 95% CI 84.4–88.6), which is even higher in the rural area (76.3, 95% CI 74.8–77.9) (6).

Unconventional production was less likely to be more adherent to the “traditional Brazilian” pattern. Conventional farmers represented the vast majority of farmers surveyed (90% of the sample, *n* = 666), which reflects the type of crop in the municipality [45]. Possibly, farmers who work with organic and agroecological farmers have different habits, determined by different work logistics, income and access to food [46].

It is also worth noting that fruit consumption was reduced among farmers (48.9% consume some kind of fruit). In Brazil, between 1987/1988 and 2008/2009, there was some stability and low levels of fruit and vegetable acquisition, including a small reduction in the consumption of vegetables [14], with lower consumption in the rural area of these two food groups [17, 19, 28].

Fruit and vegetable consumption was expected to be higher in rural areas, given the possibility of access to land and the cultivation of these products. However, the food produced by these farmers can be understood as commodities and intended for sale and income, and not perceived as self-consumption products [47]. It should be noted that, on average, subsistence production accounts for 58% of the caloric consumption of rural households, and thus 42% of the calories consumed from purchased food [48].

In accordance with this fact, the third dietary pattern detected was the “industrialized” pattern, which shows that, although farmers have a still typically local and Brazilian dietary habit, factors associated with globalization are affecting their eating habits. This, which, among other consequences, makes the contemporary rural farmer also opt for a diet with processed and ultraprocessed products [9, 21, 23, 29, 49].

Regarding the factors associated with the “industrialized” pattern, it was possible to identify an epidemiological gradient regarding age. It is worth mentioning that the younger the individual, the greater the chances of adherence to this pattern. This may show that changes in dietary patterns are occurring mainly in the younger population, as seen in rural Pelotas/RS/Brazil [29] and in Brazil as a whole [27].

In the present study, farmers from lower economic classes were found to be less likely to adhere to the “industrialized” pattern, possibly because the average value of ultra-processed foods in Brazil is higher than that of other foods [24], in contrast to what is observed in developed countries [50].

Farmers who work with temporary and permanent crops were also associated with higher adherence to this food pattern, likely due to the income proxy that this variable may present, since larger areas of land are needed for the planting of some permanent crops, such as coffee, one of the most important economic activities of the municipality [45].

Other habits of a more urbanized rural pattern could also be identified as associated with greater adherence to the third dietary pattern. The purchase of food in a variety of places, such as local shops and supermarkets, may be responsible for diversifying the purchase and consumption of products that deviate from the pattern of fresh foods produced on many farms [23, 47, 48]. In addition, higher monthly expenses on food purchases may also lead to more access to food away from home, since with the 10% increase in household income, for example, there is already a 3% increase in the share of food consumed away from home [13].

Finally, not eating at a table increased the chances of greater adherence to the industrialized pattern. It is important to remember that the family institution influences the eating habits of its members, since the practice of eating together has a significant role in learning healthy eating practices. Therefore, meals taken away from a table and away from the family unit may promote the highest consumption of ultra-processed foods [41].

Possible limitations of this study include its cross-sectional nature, which requires greater caution in interpreting the results, due to the possibility of reverse causality. In addition, the factorial analysis employed in the derivation of dietary patterns involves some subjectivity in its decision-making. However, such limitations are mitigated by detailing all deliberations [51]. It is noteworthy that the dietary patterns detected in this study were comparable with those of other studies, which validates the results obtained externally [27, 30, 34, 41–43].

## Conclusions

Despite the study being conducted in only one Brazilian municipality, the results and discussion may indicate changes in dietary patterns in rural areas of the country as a global trend. The supporting to implement and strengthen public health surveillance systems in food consumption and education for an adequate and healthy diet could be impact a large portion of the economically active population, representing by agricultural employs. Thus, measures that increase cultivation, consumption and access to traditional foods and as well as encouraging the maintenance of culinary skills, preserving the local food heritage, are considered of utmost importance and should be encouraged.

## Supplementary information

**Additional file 1.** Participant flow diagram.

## Data Availability

The datasets used and/or analysed during the current study are available from the corresponding author on reasonable request.

## Abbreviations

50p: Median
95% CI: Confidence interval of 95%
BTS: Bartlett’s test of sphericity
G: Group
IQR: Interquartile range
KMO: Kaiser-Meyer-Olkin coefficient
OR: Odds ratio
p: *p*-value
PCA: Principal Component Analysis
POF: 2008/2009 Household Budget Survey
R24h: 24-h recalls
