# Supplementary figures and images for: Dietary patterns of Brazilian farmers and their relation with sociodemographic, labor, and lifestyle conditions
Source: Nutr J. 2020 Mar 24;19:23. doi: 10.1186/s12937-020-00542-y (PMC7093971; doi:10.1186/s12937-020-00542-y)

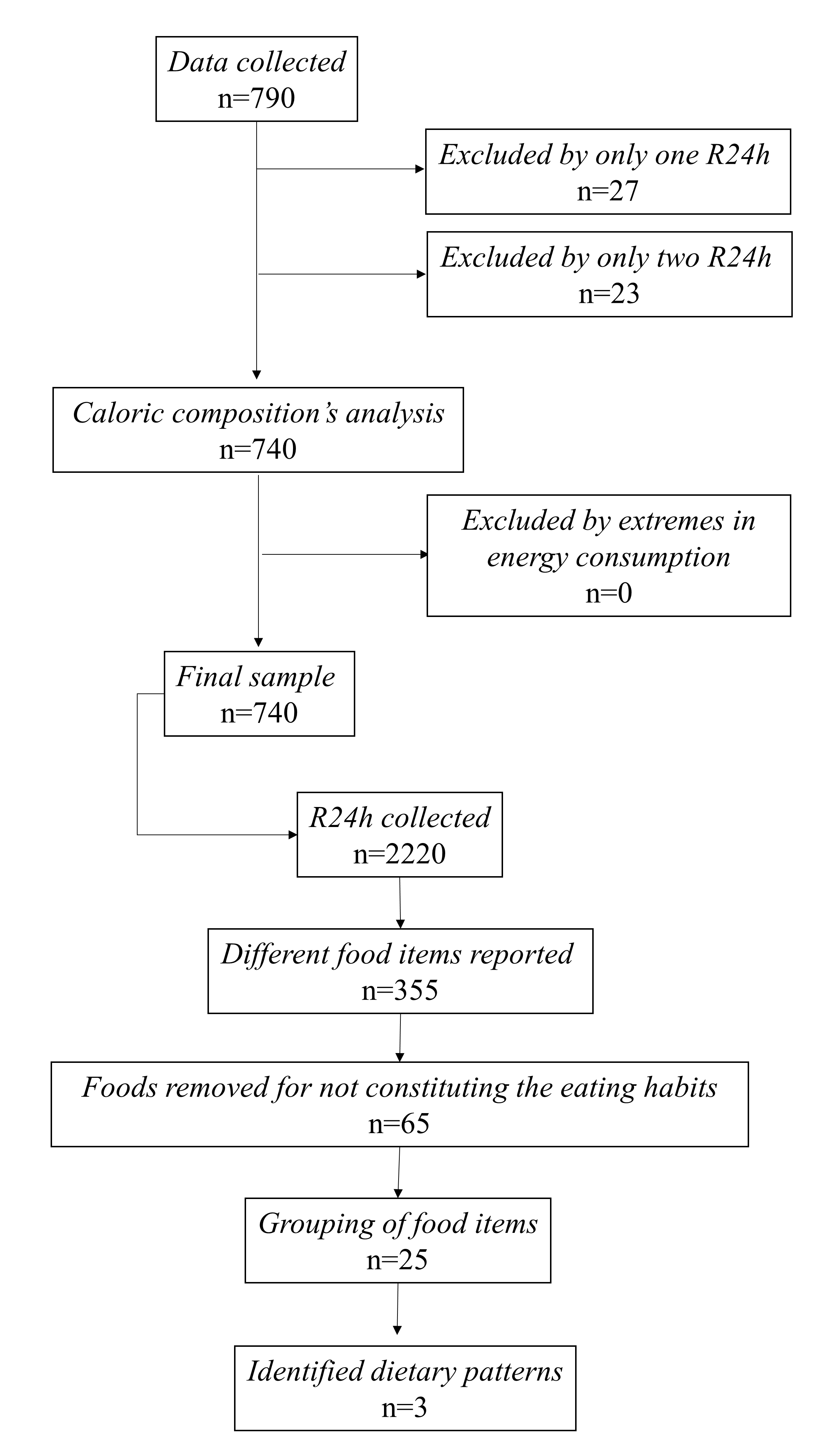

Supplement: Supplementary file 1 — Additional file 1. Participant flow diagram. [file 12937_2020_542_MOESM1_ESM.tif]
